# Global gene expression analysis of *Escherichia coli* K-12 DH5α after exposure to 2.4 GHz wireless fidelity radiation

**DOI:** 10.1038/s41598-019-51046-7

**Published:** 2019-10-08

**Authors:** Ilham H. Said-Salman, Fatima A. Jebaii, Hoda H. Yusef, Mohamed E. Moustafa

**Affiliations:** 1 Department of Biological Sciences, Faculty of Science, Beirut Arab University, Beirut, Lebanon; 2 Department of Biochemistry, Faculty of Science, Lebanese University, Beirut, Lebanon; 3 Department of Biochemistry, Faculty of Science, Alexandria University, Alexandria, Egypt

**Keywords:** High-throughput screening, Transcriptomics, Bacterial genetics, Gene ontology, Functional clustering

## Abstract

This study investigated the non-thermal effects of Wi-Fi radiofrequency radiation of 2.4 GHz on global gene expression in *Escherichia coli* K-12 DH5α. High-throughput RNA-sequencing of 2.4 GHz exposed and non-exposed bacteria revealed that 101 genes were differentially expressed (DEGs) at P ≤ 0.05. The up-regulated genes were 52 while the down-regulated ones were 49. QRT-PCR analysis of *pgaD*, *fliC*, *cheY*, *malP*, *malZ*, *motB*, *alsC*, *alsK*, *appB* and *appX* confirmed the RNA-seq results. About 7% of DEGs are involved in cellular component organization, 6% in response to stress stimulus, 6% in biological regulation, 6% in localization, 5% in locomotion and 3% in cell adhesion. Database for annotation, visualization and integrated discovery (DAVID) functional clustering revealed that DEGs with high enrichment score included genes for localization of cell, locomotion, chemotaxis, response to external stimulus and cell adhesion. Kyoto encyclopedia of genes and genomes (KEGG) pathways analysis showed that the pathways for flagellar assembly, chemotaxis and two-component system were affected. Go enrichment analysis indicated that the up-regulated DEGs are involved in metabolic pathways, transposition, response to stimuli, motility, chemotaxis and cell adhesion. The down-regulated DEGs are associated with metabolic pathways and localization of ions and organic molecules. Therefore, the exposure of *E. coli* DH5α to Wi-Fi radiofrequency radiation for 5 hours influenced several bacterial cellular and metabolic processes.

## Introduction

Electromagnetic Fields (EMF) effects on living organisms have been an important research topic for many years. Wireless fidelity (Wi-Fi) waves are part of the non-ionizing radiation of the electromagnetic spectrum. Several studies examined the non-thermal effects of high frequency electromagnetic fields of mobile phones and Wi-Fi on different strains of bacteria^1–8^. We have found previously that Wi-Fi exposure of *Escherichia coli* O157H7 increased antibiotic resistance, motility and ability to form biofilm^9^. Bacterial resistance is expanding to most commonly used antibiotics that has been considered as “global health crisis” by the World Health Organization (WHO)^10^. Flagella represent a critical virulence factor that permit bacterial motility and promote adhesion to the gastrointestinal mucins^11^. During environmental stress, bacteria produce a polysaccharide matrix and aggregate to form biofilms^12^. These virulence factors play key roles in infection initiation and development of diseases.

Gene expression in *E. coli* is influenced by environmental factors such as temperature, pH, and other stress factors^13–16^. Bacteria may activate strategies to adapt to various environmental stress. Here, we investigated the changes in global transcriptome in *E. coli* K12 DH5α after exposure to 2.4 GHz EMF emitted from a Wi-Fi router using high-throughput RNA sequencing. *Escherichia coli* K-12 DH5α strain was constructed by Douglas Hanahan and is a commonly used laboratory strain^17,18^. We used this strain as a model to understand the effects of Wi-Fi radiofrequency radiation on transcriptomes of *E. coli* bacteria.

The differences in the expression of selected genes were confirmed by RT-PCR assays.

## Results

### Transcriptome sequencing of exposed *E. coli* DH5α to Wi-Fi radiofrequency radiation

**Figure 1:**
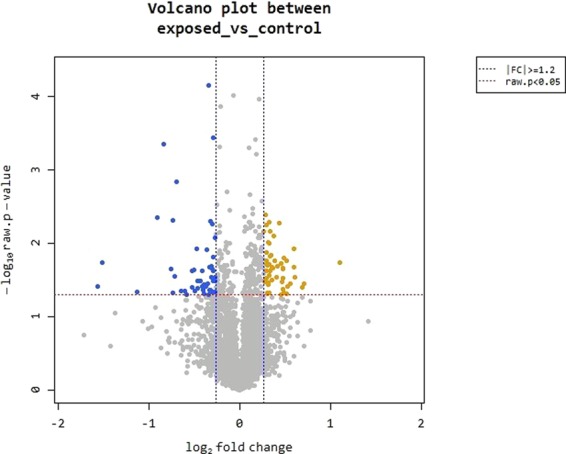
Volcano plot highlighting differentially expressed genes between unexposed and exposed bacteria. The genes are colored if they pass the thresholds for −log10 P value (P value = 0.05) and log fold change |FC| ≥1.2, yellow if they are up-regulated and blue if they are down-regulated.

The whole transcriptome sequencing was performed to study the effects of Wi-Fi exposure on global gene expression in *Escherichia coli* DH5α. RNA-seq experiments were performed in triplicates of Wi-Fi radiation exposed and non-exposed bacteria. The trimmed reads were mapped to the reference genome using Bowtie software^19^. The reads per kilobase million (RPKM) value of the genes obtained through the RNA-seq were used as the original raw data. A number of 83 genes with zero RPKMs in the 6 samples were excluded leaving 4,461 genes. During data preprocessing, low quality transcripts were filtered resulting in 4,378 genes to be studied. The distribution of gene expression between the unexposed and exposed samples is represented by the volcano plot (Fig. 1). The genes located out of the borders of the line fold change FC ≥1.2 were considered to be differentially expressed genes (DEGs). The number of DEGs at FC ≥1.2 without P-value restraint was 468, which was reduced to 101 at P < 0.05. Genes situated from the left boundary are down-regulated while those at the right are up-regulated genes. In that data set, 52 genes were significantly up-regulated and 49 genes were down-regulated after the exposure to 2.4 GHz Wi-Fi radiation.

**Figure 2:**
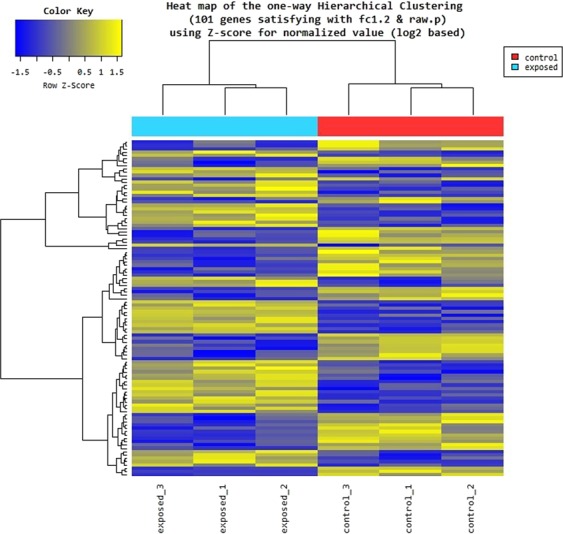
Heat map of DEGs in control and 2.4 GHz exposed bacteria (3 replicates each). Expression level is color patterned: yellow for up-regulated genes, gray for unchanged expression, and blue for down-regulated genes.

Hierarchical clustering analysis of 101 DEGs was performed to group similar samples and genes. These results were graphically depicted using heat map and dendogram (Fig. 2). Heat map shows the results of hierarchical clustering analysis which clusters genes and samples by expression level from significant list.

### Functional classification of DEGs based on gene ontology

**Figure 3:**
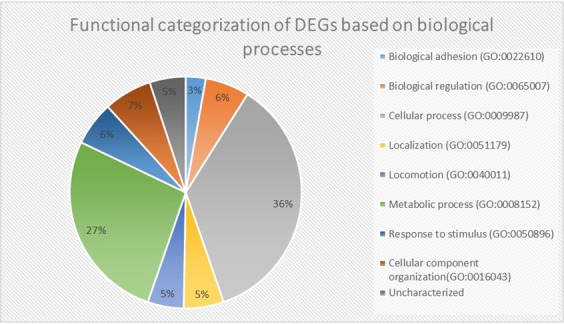
Gene ontology analysis of DEGs after exposure of E. coli DH5α to 2.4 GHz Wi-Fi.

Gene ontology (GO) analysis classifies the differentially expressed genes into three groups: biological processes, molecular functions and cellular components. The 101 DEGs at FC ≥1.2 and P-value = 0.05 were classified into eight functional groups of the biological processes category (Fig. 3). About 36% of the DEGs are implicated in cellular process, 27% in metabolic processes, 7% in cellular component organization and 6% in biological regulation. In addition, 6% of the DEGs are involved in response to stimulus, 5% in localization, 5% in locomotion and 3% in cell adhesion.

### Functional annotation clustering

Functional annotation clustering was done by DAVID tools version 6.8 to evaluate genes differentially expressed between the exposed and control bacterial cells^20,21^. Based on the enrichment score (ES), functional annotation clustering produced a total of 18 clusters from the 101 DEGs. The most enriched clusters were composed of genes involved in locomotion, localization of cell and bacterial-type flagellum-dependent cell motility (ES = 3.74) and chemotaxis and response to external stimuli (ES = 2.00). They also include genes associated with cell adhesion (ES = 1.08), cellular component organization (ES = 0.71), DNA repair and metabolism (ES = 0.63) and metabolic processes (ES = 0.53) (Table 1).

**Table 1:** Summary of functional annotation clustering analysis by DAVID tools.

Functional Annotation Cluster	Enrichment Score	Gene Count
**Annotation Cluster 1**	3.74	
Locomotion		9
Localization of cell		8
Bacterial-type flagellum-dependent cell motility		7
**Annotation Cluster 2**	2.00	
chemotaxis		5
Response to external stimulus and chemicals		8
**Annotation Cluster 3**	1.08	
Cell adhesion		4
**Annotation Cluster 4**	0.71	
Cellular component organization or biogenesis		8
**Annotation Cluster 5**	0.63	
DNA repair		4
DNA metabolism		6
**Annotation Cluster 5**	0.54	
Nucleotide biosynthetic process		5
Carbohydrate derivative metabolic process		7
Organonitrogen compound metabolic process		12

Clusters involved in the same biological process were merged together. ES threshold was set to 0.5 and gene count ≥4.

### GO enrichment analysis of DEGs

**Figure 4:**
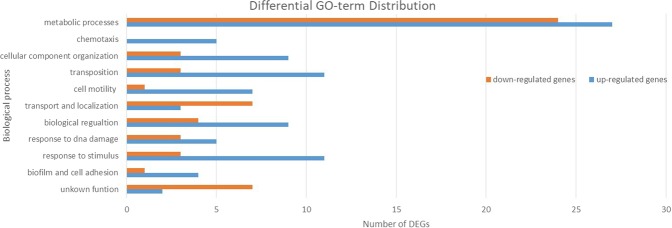
GO term enrichment analysis of up- and down-regulated differentially expressed genes.

The different functional networks for down-regulated and up-regulated genes are shown in Fig. 4. Among the 52 up-regulated genes 50 were assigned to GO functions. The 49 down-regulated genes contained 42 with GO functions. The other 9 genes in the DEGs were coding for t-RNA or have uncharacterized functions. The entire list of the up-regulated and down-regulated genes can be found as Supplementary Tables S1 and S2. The higher percentage of DEGs are implicated in metabolic processes. The up-regulated DEGs are involved in cell motility, cell adhesion, chemotaxis, transposition, cellular component organization, response to stimulus and DNA damage. Most of the down-regulated DEGs are associated with transport and localization of nitrogen compounds, organic substances and ions (Fig. 4).

### KEGG pathways analysis

Kyoto encyclopedia of genes and genomes (KEGG) pathways were obtained within the analysis by DAVID^22^. KEGG Pathways affected by the exposure of 2.4 GHz Wi-Fi radiation are: flagellar assembly, bacterial chemotaxis and two-component system.

**Figure 5:**
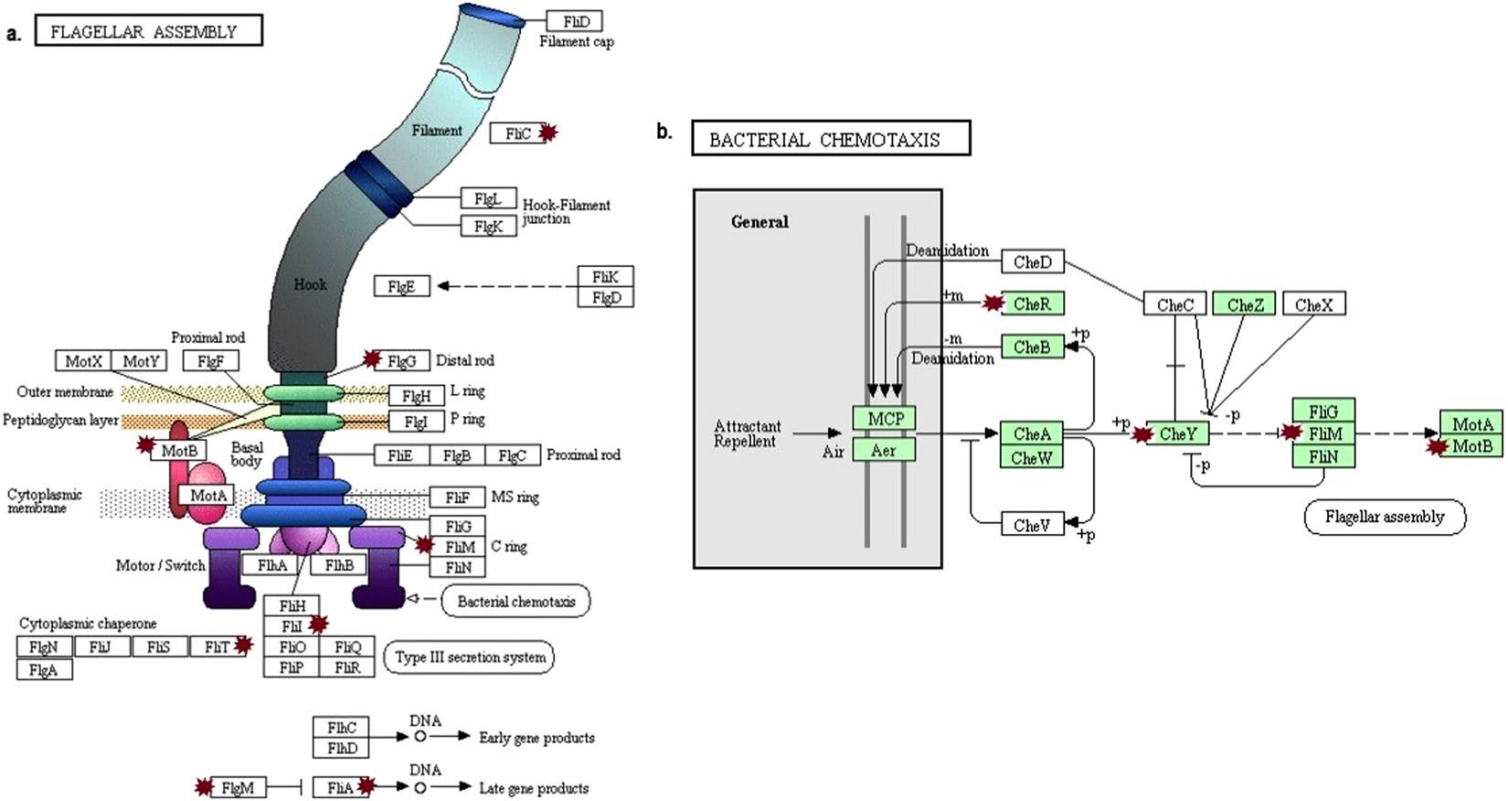
Flagellar pathway assembly and bacterial chemotaxis from KEGG database. Red stars point to the up-regulated genes affected in (**a**) the flagelar assembly pathway (ko02040). (**b**) the bacterial chemotaxis pathway (ko02030). Some of the affected genes belong to the two-component system pathway (ko02020).

Figure 5 shows the genes involved in the synthesis, assembly of flagella and chemotaxis. The genes *flgG, motB, fliM, fliL, fliT, fliC, fliA, flgM*, *cheR* and *cheY* were up-regulated by the exposure to Wi-Fi waves. FLgG is a protein part of the flagellar basal-body which connects to components of the flagellar motor. MotB and FliM are elements of the flagellar motor required for the rotation of the flagellar^23^. FliL controls the rotational direction of flagella during chemotaxis. FliT acts as an export chaperone for the filament-capping protein FliD. The *fliC* gene code for the subunit protein flagellin which is polymerized to form the filaments of bacterial flagella. FliA (σ28) and FlgM (anti- σ28) control the expression of late flagella-related genes^24^. CheR is responsible for the methylation of the membrane-bound methyl-accepting chemotaxis proteins and CheY is implicated in the transduction of signals from the chemoreceptors to the flagellar motors^23^.

**Figure 6:**
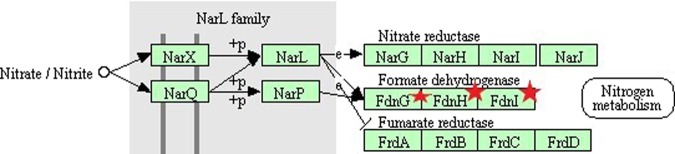
Two-component system pathway from KEGG database (ko02020). Red stars point to the down-regulated genes affected in the nitrogen metabolism.

Figure 6 shows the genes involved in nitrogen metabolism. The genes *fdnG, fdnI*, and *fdnH* are down-regulated by the exposure to Wi-Fi radiation and are part of the two-component system pathway. These genes code for the subunits of the protein formate dehydrogenase^25^. During anaerobic respiration, *E. coli* utilize formate as electron donor and nitrate as electron acceptor through formate dehydrogenase^26^.

### Validation of several DEGs by quantitative RT-PCR

**Figure 7:**
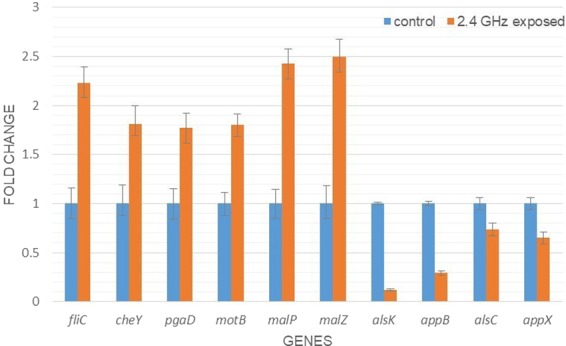
Quantitative real time PCR chart; relative expression level normalized with 2 housekeeping genes: gyrA and frr.

We measured the expression of representative DEGs by qRT-PCR. These DEGs examined included *pgaD, fliC, cheY, malP, malZ, motB, alsC, alsK, appB* and *appX*. The qRT-PCR results were analyzed by REST 2009 software (Fig. 7). The housekeeping genes *gyrA* and *frr* were used for gene normalization. The results from qRT-PCR assays were found to be consistent with that obtained from RNA-sequencing; P < 0.05 (Table 2).

**Table 2:** Comparison of fold change difference between RNA-seq and qRT-PCR.

Gene	RNA-seq		qRT-PCR	
	*FC	p-value	**FC [std error]	p-value
*pgaD*	1.78	0.0329	1.775[1.612 to 1.924]	<0.001
*fliC*	2.12	0.0396	2.236[2.085 to 2.399]	<0.001
*cheY*	1.76	0.0348	1.813 [1.692 to 2.001]	<0.001
*malP*	2.4	0.0286	2.426[2.275 to 2.573]	0.024
*malZ*	2.5	0.0382	2.495[2.339 to 2.674]	0.039
*motB*	1.69	0.0355	1.804 [1.743 to 1.915]	0.04
*alsC*	−1.42	0.0388	−1.35[−1.48 to −1.26]	<0.001
*alsK*	−8.78	0.0459	−8.33 [−9 to −7.63]	<0.001
*appB*	−2.47	0.0473	−3.38 [−3.69 to −3.15]	0.036
*appX*	−1.5	0.0183	−1.53[−1.7 to −1.41]	0.033

^*^FC: fold change.

**[std error]: standard error interval.

## Discussion

EMF exposure induced different responses in bacteria depending on frequency, intensity, exposure time and the organism model^27^. Two hours of mobile phone exposure revealed no effects on the growth and susceptibility to different antibiotics *of S. aureus, S. epidermis*, and *P. aeruginosa*^8^. The exposure of *S. aureus* to mobile phone for one hour did not affect their ability to form biofilm^3^. Longer time of GSM and Wi-Fi exposure affected the growth, antibiotic susceptibility and biofilm formation of *P. aeruginosa, E. coli, S. aureus, S. epidermis* and *L. monocytogenes*^6,7,9^. The prolonged EMF exposure resulted in major effects in *K. pneumonia* before they reach an adaptation stage^5^. Some bacteria with resistance to multidrugs were detected in the vicinity of telecommunication stations^28^. Furthermore, the microbial growth of human skin microbiota was disrupted after exposure to radiofrequency EMF (RF-EMF)^29^.

The WHO recommended evaluation of the biological effects of existing EMF exposure in a population before providing permission for establishment of new EMF networks^30,31^. Understanding the mechanisms by which RF-EMF influences human health is also important to reduce the risk of incidence of diseases^32^.

In this study, most of the DEGs in DH5α cells exposed to Wi-Fi waves are involved in cellular and metabolic processes. The upregulated *sulA, yjjQ, oxC* and *arsC* genes are associated with stress response. The *sulA, yadC, sbcB, recT* and *arsC* genes are part of the defense system against DNA damage. SulA in *E. coli* is induced by the SOS response to DNA damage resulting in an inhibition of cell division by interaction with tubulin-like FTsZ. At the site of cell division, FtsZ assembles into a ring which is essential for bacterial cell division^33^. In parallel, a reduction of *ZipA*, coding for a stabilizer protein of FtsZ, was detected. This regulatory network elucidate morphological changes observed after exposure to extremely low frequency EMF (ELF-EMF) by affecting cell division^34,35^. Only 3 of the genes associated with response to DNA damage, *udp*, *purN* and *yafO*, were down-regulated.

The up-regulated *ampE, yadC* and *ybhG* genes are related to antibiotics response. AmpE is implicated in the expression of β-lactamase and plays a role in peptidoglycan murein recycling^36,37^. YadC is a fimbrial-like protein that is induced immediately after exposure to stress and provide antimicrobial protection^38^. YbhG is involved in the sensitivity control to chloramphenicol through the efflux pathway^39^. These results confirm the data of some previous studies indicated altered antibiotic resistance after exposure to RF-EMF^5,6,9^.

Our data showed that genes involved in chemotaxis and motility *such as fliA, flgM, motB, fliC, cheY, cheR, fliM, fliL, flgG* and *fliT* were up-regulated by the exposure to Wi-Fi waves. Overexpression of CheY in association with MotA and MotB increases motility^40^. These results confirm our prior finding that Wi-Fi radiation enhanced motility of *E. coli* O157 H7 by 28%^9^, which may be a strategy of the pathogen for survival after excessive exposure to radiation stress. *E. coli* O157H7 cells motility was increased under different stress conditions such as heat and acid^41,42^. Motility of *E. coli* is important for starting biofilm formation^43^. We have previously reported that Wi-Fi radiofrequency radiation triggers biofilm formation in *E. coli*^9^. Our data indicated that the exposure to Wi-Fi radiofrequency radiation induced the expression of *pagD, yadM, yadC* and *fimI* involved in cell adhesion and biofilm formation. The down-regulated genes included *yadV* which codes for a fimbrial chaperone involved in cell adhesion and *yciG* that is implicated in flagellum-dependent swarming motility. The changes in bacterial gene expression permit the survival in environments with rapidly changing conditions. Therefore, biofilm formation is a sort of bacterial defense against stressful conditions.

Gene ontology analysis revealed that the down-regulated DEGs are involved mostly in metabolic, cellular process and localization. For example, the *ssuA, ppdB, xanP, gadC, ompL, sgcB, yacD* and *alsC* genes are associated with localization and transport of different compounds. The *ompL, alsC* and *sgcB* genes have a role in carbohydrate transport. The *ompL* and *gadC* genes facilitate ions transport. The *gadC, xanP* and *ppdB* genes are related to nitrogen compound transport. In addition, the down-regulated *pyrI, purE, pyrB* and *purN* genes are involved in nucleotide biosynthetic pathway. We observed down-regulation of the *appX* and *appB* genes, which are part of cytochrome bd-II oxidase and are involved in aerobic electron transport chain^44^. Moreover, down-regulation of the *ldrA, ldrB* and *ldrC* genes type I toxin-antitoxin system that inhibits cell growth, were detected^45^. Many genes involved in anaerobic respiration were down-regulated. For instance, the down-regulated *fdnI, fdnH* and *fdnG* genes belongs to the two-component system pathway and plays a role in nitrogen metabolism^25^. The down-regulated *hyaA, hyaB* and *hyaD* genes are involved in fermentation. *hya* expression is affected by nutrients starvation and external pH and hence it is involved in response to stress^46^.

As consequence of regulatory network, we detected increased expression of *yhdX and agaC* genes that play a role in transport of amino acids and sugar, respectively. The up-regulated *puuC, eutP, metC, kbaY* genes are involved in metabolic pathways. In addition, we observed up-regulation of *malP* and *malZ genes*, part of the maltodextrin system, which are involved in the uptake of maltose by ABC transporter^47^.

We found that 11 of the up-regulated and 3 of the down-regulated genes are associated with bacterial transposition activity. Transposases increase genomic rearrangements by incorporation of adjacent genes during transpositions^48^. Nutritional and cellular stress as well as environmental adaptation trigger transposition in *Escherichia coli*^49,50^. It has been reported that extremely low frequency EMF affected transposition activity in *E. coli*^51^.

Microorganisms often switch between phases of growth and non-growth to adapt to environmental conditions and they can enter dormant state. In this viable but non-culturable state, bacteria can still preserve certain metabolic activity but are incapable to divide due to their minor adaptation capacity^52^. Alterations in bacterial gene expression allow bacteria to resist the stress conditions. Our data showed that both genes involved in aerobic and anaerobic respiration were down-regulated after exposure to Wi-Fi radiofrequency radiation. Decreased metabolic activity in bacteria has been linked with increased persistence. Part of the bacterial population enters into dormant state through metabolic inactivity and growth arrest in order to survive in the stress conditions^53^. Other persistence mechanisms are also found in *E. coli* such as SOS response, DNA repair, pump efflux and biofilm formation^52,54,55^. On the other hand, the exposure of bacteria to EMF can be valuable when interfering with these phenomena and may compromise therapeutic success. It has been shown that the exposure to ELF-EMF resulted in antibacterial effects as well as altered bacterial morphology and decreased the viability in biofilms suggesting a reduced persistence^35,56,57^. Further research is required to clarify the effects of EMF on their persistence and therapeutic success.

**Figure 8:**
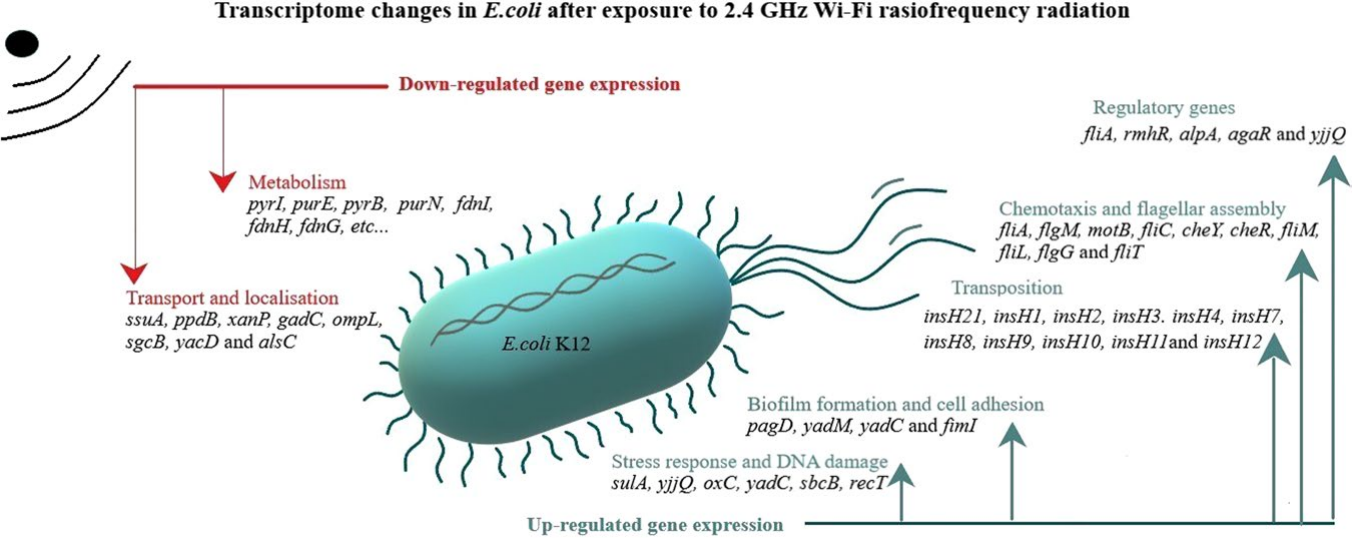
Proposal hypothesis for the mechanisms by which 2.4 GHz EMF influences the bacterial transciptome of *E. coli* K12.

The changes in bacterial gene expression in response to exposure to Wi-Fi waves are summarized in Fig. 8. Our results showed alterations in the transcription of genes involved in metabolism, transport, flagellar assembly, response to DNA damage, transposition and biofilm formation. In addition, there was increased transcription of five regulatory genes *agaR, yjjQ, alpA, rmhR* and *fliA* after exposure to Wi-Fi waves. agaR is a repressor that regulate *aga* operon which is related to transportation and degradation of N-acetylgalactosamine^58^. The transcriptional regulator YjjQ is part of the LuxR family and play a role in detoxification of methylglyoxal^59^. AlpA is a transcriptional regulator of *intA* expression which results in excision of the cryptic prophage CP4-57 and play a role in biofilm formation^60,61^. RmhR is a transcriptional factor part of the *rhm* operon involved in L-rhamnonate utilization^62^. Moreover, sigma factor FliA (σ28) control the expression of late flagella-related genes^24^. Therefore, the exposure of *E. coli* to Wi-Fi radiofrequency radiation influenced the transcription of a network of regulatory genes that affected several biological processes.

Several studies investigated the effects of exposing human and rodent cells to Wi-Fi radiofrequency radiation on gene expression. Exposure of human cells HL-60 to 2.4 GHz waves for 2 h altered the expression of 221 genes. The up-regulated ones were associated with stress response and apoptosis while the down-regulated genes were involved in cell cycle, metabolism and transport^63^. Moreover, the exposure of rat hippocampus to Wi-Fi radiation for 12 hours influenced the expression of 41 genes related to heat shock proteins, metabolism and signal transduction^64^. Other studies showed that long term exposure to Wi-Fi waves changed microRNAs levels and gene expression in rat brain^65,66^.

In summary, the exposure of *E. coli* DH5α to Wi-Fi radiofrequency radiation for 5 hours up-regulated 52 genes and down-regulated 49 genes. The expression of detected genes was confirmed by qRT-PCR assays. Gene ontology analysis showed that the higher percentage of DEGs are implicated in cellular and metabolic processes. Most of up-regulated DEGs plays a role in transposition, response to stimuli, motility and chemotaxis while the down-regulated DEGs are mainly associated with transport and localization of nitrogen compounds, organic substances and ions. DAVID functional clustering indicated that DEGs with high enrichment score for localization of cell and bacterial-type flagellum-dependent cell motility was 3.74. The ES for chemotaxis and response to external stimulus was 2 and for cell adhesion was 1.08. KEGG pathways analysis revealed that the pathways for flagellar assembly, bacterial chemotaxis and two-component system were affected after the exposure to 2.4 GHz Wi-Fi radiation. Our results showed that the exposure to these waves influenced transcriptomes responsible for metabolic and cellular processes, localization, stress response, transposition, motility, chemotaxis and cell adhesion.

This is the first report investigating the alterations in the bacterial transcriptome profiling after exposure to Wi-Fi radiofrequency radiation. Detailed information of RNA-seq analysis following Wi-Fi radiation exposure in *E. coli* could be valuable to understand the effects of Wi-Fi radiation on pathogenic traits of bacteria, particularly antibiotic resistance, motility and biofilm formation. The results of this study open the door for further investigation of the mechanisms of effects of RF-EMF on pathogenic and non-pathogenic bacteria that may have influence in human health and disease. Further studies are required to explore deeply the mechanisms by which 2.4 GHz EMF influences the bacterial transcriptome. Undervaluing the problem of telecommunication exposure could cause further rise in infectious diseases or their complications.

## Materials and Methods

### Microwave exposure system

**Figure 9:**
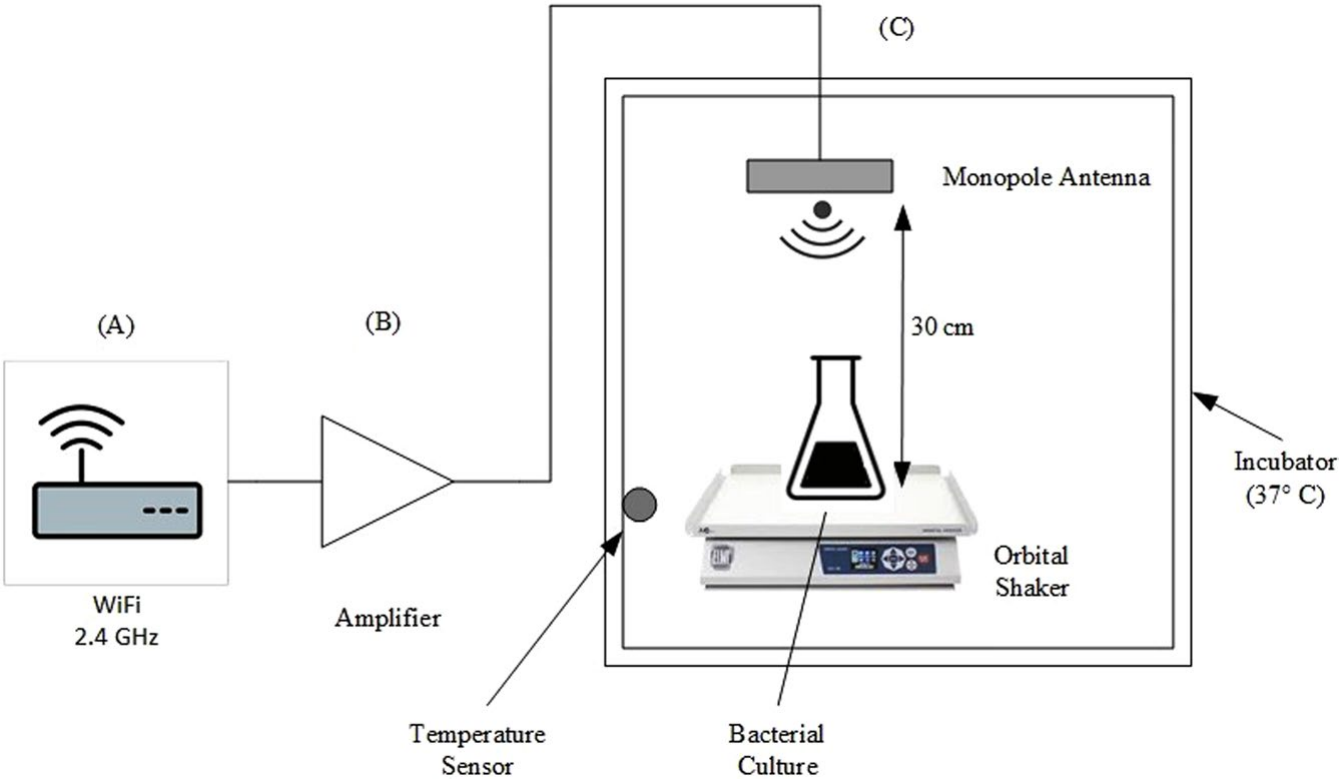
Schematic flow chart of the Wi-Fi radiofrequency exposure model. (**A**) Wi-Fi router (**B**) amplifier (**C**) monopole antenna mounted in an incubator chamber with temperature control.

Wi-Fi radiofrequency radiation was generated by a wireless router extended range (TL-WR524G-Tp-Link -China) corresponding to 2.4 GHz frequency, connected to an amplifier and monopole antenna. The mounted system was placed in an incubator at 30 cm from the bacterial culture. *E. coli* cells were continuously exposed to Wi-Fi radiation for 5 hours (Fig. 9). The control bacteria were placed in Faraday bags to their exposure to limit any exterior radiation.

### Bacterial growth conditions

*E. coli* K12 DH5α transformed with puc18 plasmid (Sigma-Aldrich-Germany) were cultured in Luria Bertani (LB) medium with 100 μg/ml ampicillin (Sigma-Aldrich-Germany) under aerobic conditions. Overnight cultures were inoculated at a dilution of 1:100 into a new LB medium with ampicillin and were incubated at 37 °C with shaking at 200 rpm until they reached mid-exponential phase (OD600 = 0.6). Bacteria protect reagent (Qiagen-Germany) was added and the bacterial cultures were centrifuged at 4000 rpm for 10 min. The bacterial pellets were collected for extraction of total RNA followed by next generation sequencing and quantitative real-time PCR (qRT-PCR) assays.

### Extraction of total RNA and high throughput RNA sequencing

Total RNA was extracted from the Wi-Fi exposed and control bacteria by the bacteria protect-RNeasy kit (QIAGEN-Germany). Any remaining genomic DNA was digested by On-column RNase-Free DNase kit (Qiagen-Germany). The concentration of RNA was measured by a NanoDrop 2000 (Thermo Fisher Scientific- USA). Triplicates of RNA samples with RNA integrity number >7 were used for high throughput sequencing by Macrogen Next Generation Sequencing Division (Macrogen, Seoul, South Korea). Libraries were generated using Ribo-Zero rRNA Removal Kit (Bacteria) and TruSeq RNA Sample Prep (Illumina-USA). Sequencing was performed by NovaSeq. 6000 System (Illumina-USA) according to the user guide (Document #1000000019358). Bowtie aligner 1.1.2 was used to map sequence reads against the GCF_002848225.1_ASM284822v1 genome reference and read numbers were counted using the HTSeq version 0.10.0^19^. Expression profile was calculated for each sample and gene as read count. Differentially expressed genes (DEGs) analysis of the 2.4 GHz exposed bacteria compared to the unexposed bacteria was performed on a comparison pair (exposed vs. control) using reads per kilobase million (RPKM). Genes with a fold change |FC| ≥1.2 and p-value < 0.05 between the two samples were considered as DEGs. The RNA-seq data obtained from this study has been deposited in the NCBI’s Gene Expression Omnibus and is accessible through GEO Series accession number GSE126584^67^.

### Quantitative real-time PCR

cDNA was synthesized by the FIRE Script RT cDNA synthesis kit (Solis Biodyne-Estonia). qRT-PCR was performed using Rotor-Gene Q (QIAGEN-Germany). Each qRT-PCR reaction contained 1 uL cDNA, 5 μL QuantiTect SYBR Green PCR Master mix (Qiagen), and 0.5 μM specific primers. The reaction cycle was: denaturation at 95 °C for 5 min; 45 cycles of denaturation at 95 °C for 10 s, annealing temperature for 30 s and extension at 72 °C for 20 s. The annealing temperatures were experimentally determined using direct PCR. Primers are listed in Table S3 (Supplementary Information).

### Data analysis of relative target gene expression

QRT-PCR efficiencies were measured from the slope of a linear regression model for each pair of primers where the reaction efficiency (E) = 10(−1/slope)^68,69^. The calibration curve was determined by the Ct with serial cDNA concentrations. The results were analyzed by the Rotor gene Q v. 2.3.1 software (QIAGEN-Germany). The relative expressions of *pgaD, fliC, cheY, malP, malZ, motB, alsC, alsK, appB* and *appX* were analyzed using Relative Expression Software Tool (REST 2009), taking in consideration the reaction efficiency and reference gene normalization using Pfaffl method^68,69^ (https://www.gene-quantification.de/rest-2009.html). The results were normalized to the levels of the two housekeeping genes (*DNA gyrase subunit A* (*gyrA*) and *ribosome-recycling factor* (*frr*)). Statistical significant difference was considered at P ≤ 0.05.

### Gene ontology and clusters analysis

Database for annotation, visualization and integrated discovery (DAVID) online tool was used to find out the biological processes of gene ontology (GO) for the differentially expressed genes (http://david.abcc.ncifcrf.gov/)^20,21^. The functional annotation clustering and Kyoto encyclopedia of genes and genomes (KEGG) pathways were obtained within the analysis by DAVID^22^. Enrichment score (ES) threshold was fixed to 0.5, and clusters involved in the same biological process were combined. To analyze the co-expression network between the up-regulated and down-regulated DEGs, enrichment analysis was done using UniProt GO Annotation^70^. For clarity GO classifications by biological function were manually grouped into sub-categories.

## Supplementary information

Table S1,Table S2, Table S3
