# Supplementary material for: Global gene expression analysis of Escherichia coli K-12 DH5α after exposure to 2.4 GHz wireless fidelity radiation
Source: Sci Rep. 2019 Oct 8;9:14425. doi: 10.1038/s41598-019-51046-7 (PMC6783421; doi:10.1038/s41598-019-51046-7)
Supplement: Supplementary file 1 — Table S1,Table S2, Table S3 [file 41598_2019_51046_MOESM1_ESM.pdf]

Global gene expression analysis of Escherichia coli K-12 DH5α after exposure to 2.4 GHz wireless fidelity radiation.
Ilham H. Said-Salman, Fatima A. Jebaii, Hoda H. Yusef, Mohamed E. Moustafa.

Table S1. Up-regulated genes after exposure to 2.4 GHz Wi-Fi for 5 hours. A single gene may be implicated in more than one GO process.

Gene names	Putative function	**GO term**
cheR	Chemotaxis, protein methylation	**Chemotaxis (GO:0006935)**<br>**Metabolic process (GO:0008152)**
cheY	Chemotaxis, protein acetylation<br>Signal transduction system	**Bacterial-type flagellum-dependent cell motility (GO:0071973)**<br>**Chemotaxis (GO:0006935)**<br>**Biological regulation (GO:0065007)**<br>**Metabolic process (GO:0008152)**
motB	Motility protein B, chemotaxis	**Bacterial-type flagellum-dependent cell motility (GO:0071973) and chemotaxis (GO:0006935)**
fliC	Flagellin	**Bacterial-type flagellum-dependent cell motility (GO:0071973)**
fliT	Flagellar protein, negative regulation of bacterial-type flagellum assembly, flagellum organization, protein folding	**Bacterial-type flagellum-dependent cell motility (GO:0071973)**<br>**Biological regulation (GO:0065007)**
fliL	Flagellar protein, chemotaxis	**bacterial-type flagellum-dependent swarming motility (GO:0071973)**<br>**Chemotaxis (GO:0006935)**
fliM	Flagellar motor switch protein, chemotaxis	**Bacterial-type flagellum-dependent cell motility (GO:0071973)**<br>**Chemotaxis (GO:0006935)**
flgG	Flagellar basal-body rod protein	**Bacterial-type flagellum-dependent cell motility (GO:0071973)**
fimI	Fimbrin-like protein, pilus organization	**Cell adhesion and biofilm (GO:0043709)**
pgaD	Biofilm PGA synthesis protein	**Cell adhesion and biofilm (GO:0043709)**
yadC	Fimbrial-like protein, response to DNA damage, pilus organization, response to antibiotic	**Cell adhesion and biofilm (GO:0043709)**<br>**Response to stimulus (GO:0050896)**
yadM	Fimbrial-like protein, pilus organization	**Cell adhesion and biofilm (GO:0043709)**
insH21	Transposase activity, Involved in the transposition of the insertion sequence IS5.	**Transposition, DNA- mediated (GO:0006313), Metabolic process (GO:0008152)**
insH1	Transposase activity, Involved in the transposition of the insertion sequence IS5.	**Transposition, DNA- mediated (GO:0006313), Metabolic process (GO:0008152)**

insH2	Transposase activity, Involved in the transposition of the insertion sequence IS5.	**Transposition, DNA- mediated (GO:0006313), Metabolic process (GO:0008152)**
insH3	Transposase activity, Involved in the transposition of the insertion sequence IS5.	**Transposition, DNA- mediated (GO:0006313), Metabolic process (GO:0008152)**
insH4	Transposase activity, Involved in the transposition of the insertion sequence IS5.	**Transposition, DNA- mediated (GO:0006313), Metabolic process (GO:0008152)**
insH7	Transposase activity, Involved in the transposition of the insertion sequence IS5.	**Transposition, DNA- mediated (GO:0006313), Metabolic process (GO:0008152)**
insH8	Transposase activity, Involved in the transposition of the insertion sequence IS5.	**Transposition, DNA- mediated (GO:0006313), Metabolic process (GO:0008152)**
insH9	Transposase activity, Involved in the transposition of the insertion sequence IS5.	**Transposition, DNA- mediated (GO:0006313), Metabolic process (GO:0008152)**
insH10	Transposase activity, Involved in the transposition of the insertion sequence IS5.	**Transposition, DNA- mediated (GO:0006313), Metabolic process (GO:0008152)**
insH11	Transposase activity, Involved in the transposition of the insertion sequence IS5.	**Transposition, DNA- mediated (GO:0006313), Metabolic process (GO:0008152)**
insH12	Transposase activity, Involved in the transposition of the insertion sequence IS5.	**Transposition, DNA- mediated (GO:0006313), Metabolic process (GO:0008152)**
oxc	Response to acidic pH, oxalate catabolic activity	**Response to stimulus (GO:0050896), Metabolic process (GO:0008152)**
yjjQ	Response to methylglyoxal, Regulation of transcription in response to methylglyoxal	**Response to stimulus (GO:0050896)**<br>**Biological regulation (GO:0065007)**
arsC	Response to DNA damage stimulus; response to arsenic, arsenate reductase activity	**Response to stimulus (GO:0050896)**<br>**Metabolic process (GO:0008152)**
sulA	Cellular response to DNA damage stimulus; SOS response, Negative regulation of cell division	**Response to stimulus (GO:0050896)**<br>**Biological regulation (GO:0065007)**
sbcB	DNA repair, Putative phosphotransferase enzyme IIB component	**Response to stimulus (GO:0050896)**<br>**Transport (GO:0006810)**
recT	DNA renaturation, DNA synthesis Involved in double-strand break repair via homologous recombination	**Response to stimulus (GO:0050896) Metabolic process (GO:0008152)**
ybhG	Response to antibiotic	**Response to stimulus (GO:0050896)**
ampE	Response to antibiotic	**Response to stimulus (GO:0050896)**

fliA	Sigma factor responsible for initiation of transcription of genes involved in motility and flagellar synthesis	**Biological regulation (GO:0065007)**
flgM	Anti-sigma factor FliA , negatively regulates flagellar genes	**Biological regulation (GO:0065007)**
puuC	Putrescine catabolic process	**Metabolic process (GO:0008152)**
eutP	Ethanolamine catabolic process	**Metabolic process (GO:0008152)**
metC	Methionine biosynthetic process	**Metabolic process (GO:0008152)**
kbaY	Carbohydrate metabolic process	**Metabolic process (GO:0008152)**
sgcB	Kinase activity	**Metabolic process (GO:0008152)**
malP	Glycogen catabolic process	**Metabolic process (GO:0008152)**
malZ	Oligosaccharide catabolic process	**Metabolic process (GO:0008152)**
rhmR	Transcription factor activity	**Biological regulation (GO:0065007)**
alpA	Biofilm formation<br>Prophage CP4-57 regulatory protein	**Biological regulation (GO:0065007)**
ykfJ	Pseudogene	**RNA processing (GO:0006396)**
agaR	Repressor for the aga operon for N-acetyl galactosamine transport and metabolism.	**Biological regulation (GO:0065007)**
yhdX	Putative amino-acid ABC transporter permease protein	**Transport (GO:0006810)**
agaC	N-acetylgalactosamine permease IIC component 1	**Transport (GO:0006810)**
yehC	Chaperone-mediated protein folding	**Cell wall organization (GO:0071555)**
yehR	Lipid-anchor	**Plasma membrane (GO 0005886)**
nlpA	Lipid-anchor, plasma membrane methionine transport	**Plasma membrane (GO 0005886)**
essQ	Prophage lysis protein	**cytolysis (GO:0019835)**

Table S2. Down-regulated genes after exposure to 2.4 GHz Wi-Fi for 5 hours. A single gene may be implicated in more than one GO process.

Gene names	Putative function	**GO term**
alsC	Carbohydrate transport	**Localization and transport** (**GO:0051179)**
gadC	Amino acid transport, maintain a near-neutral intracellular pH	**Localization and transport (GO:0051179)**<br>**Biological regulation(GO:0065007)**
ompL	Oligosaccharide and ion transport	**Localization and transport** (**GO:0051179)**
ppdB	Protein secretion by the type II secretion system	**Localization and transport** (**GO:0051179)**
ssuA	Alkanesulfonate transport, Sulfur compound metabolic process	**Localization and transport** (**GO:0051179)**<br>**Metabolic process (GO:0008152)**
xanP	Xanthine transport	**Localization and transport** (**GO:0051179)**

ycaD	transmembrane transport	**Localization and transport (GO:0051179)**
ytfT	transmembrane transport	**Localization and transport (GO:0051179)**
insL1	Involved in the transposition of the insertion sequence IS186.	**Transposition, DNA-mediated (GO:0006313) Metabolic process (GO:0008152)**
insL2	Involved in the transposition of the insertion sequence IS186.	**Transposition, DNA-mediated (GO:0006313) Metabolic process (GO:0008152)**
insL3	Involved in the transposition of the insertion sequence IS186.	**Transposition, DNA-mediated (GO:0006313) Metabolic process (GO:0008152)**
pyrI	Pyrimidine nucleotide biosynthetic process	**Metabolic process (GO:0008152)**
purE	'De novo' IMP biosynthetic process	**Metabolic process (GO:0008152)**
rimO	Peptidyl-L-beta-methylthioaspartic acid biosynthetic process from peptidyl-aspartic acid; tRNA modification	**Metabolic process (GO:0008152)**
cobC	Cobalamin biosynthetic process	**Metabolic process (GO:0008152)**
fdnI	Anaerobic respiration; formate oxidation; respiratory electron transport chain	**Metabolic process (GO:0008152)**
fdnH	Anaerobic respiration; formate oxidation; respiratory electron transport chain	**Metabolic process (GO:0008152)**
fdnG	Anaerobic respiration; formate oxidation; respiratory electron transport chain	**Metabolic process (GO:0008152)**
alsK	D-allose catabolic process	**Metabolic process (GO:0008152)**
appB	Aerobic electron transport chain; oxidative phosphorylation	**Metabolic process (GO:0008152)**
hyi	Glyoxylate metabolic process	**Metabolic process (GO:0008152)**
eutQ	Ethanolamine catabolic process	**Metabolic process (GO:0008152)**
hyaB	Anaerobic respiration; fermentation	**Metabolic process (GO:0008152)**
hyaA	Anaerobic respiration; fermentation	**Metabolic process (GO:0008152)**
hyaD	Protein processing	**Metabolic process (GO:0008152)**
pyrB	'De novo' pyrimidine nucleobase biosynthetic process; cellular amino acid metabolic process; nitrogen compound metabolic process	**Metabolic process (GO:0008152)**
purN	'De novo' IMP biosynthetic process, cellular response to DNA damage stimulus	**Metabolic process (GO:0008152)**<br>**Response to stimulus (GO:0050896)**
udp	Uridine catabolic process, cellular response to DNA damage stimulus	**Metabolic process (GO:0008152)**<br>**Response to stimulus (GO:0050896)**
lpp	Lipid modification	**Metabolic process (GO:0008152)**
yafO	SOS response, Negative regulation of translation, DNA repair	**Response to stimulus (GO:0050896)**<br>**Biological regulation(GO:0065007)**

		**Metabolic process (GO:0008152)**
yiiE	Regulation of transcription, DNA-templated	**Biological regulation(GO:0065007)**
yadV	Probable fimbrial chaperone	**Cell adhesion (GO:0071555)**
yciG	Flagellum-dependent swarming motility	**Bacterial-type flagellum-dependent swarming motility (GO:0071978)**
yidG	Inner membrane protein	**integral component of membrane (GO:0016021)**
yddB	Pseudogene	**integral component of membrane (GO:0016021)**
appX	Part of cytochrome bd-II oxidase	**Plasma membrane (GO:0005886)**
zapA	Activator of cell division	**Cell division (GO:0000917)**
ldrA	Toxic component	**Plasma membrane (GO:0005886)**
ldrB	Toxic component	**Plasma membrane (GO:0005886)**
ldrC	Toxic component	**Plasma membrane (GO:0005886)**
yqhh	Peptidoglycan binding, cell outer membrane	**intrinsic component of cell outer membrane (GO:0031230)**

Table S3. QRT-PCR primer sets used in this study and their target genes.

Gene name	Forward primer	Reverse primer	Amplicon size
pgaD	TTACGACCCGACAATCACCAG	GCCAGTAATAACCCGTCAGCA	123
fliC	ATTCCGTTCTTCCCTCGGTG	TGGACACTTCGGTCGCATAG	131
cheY	AGTGCGTAACCTGCTGAAAGA	AAATCCATAACCGCCTGCCT	100
malP	TCGGCTATCGTAATGGCGTG	TTCGGCACGCAAGAAATCAC	107
malZ	CAGGTGCTGTATGCGGAAGA	CACGGCATTGAGAAACGGTG	132
motB	GAACTGATGGTCGGAGGGTT	GCCTGTTCGGCTTGTTTGTTC	146
alsC	AACTGATGTTGGCAGGTGTTG	AGGGTGATGATGAACGGGTG	130
alsK	CGATCTTTTCGTCCATGCGG	GAAGGCGGGCATATCCATCA	142
appB	CGACGGATTTGACATGGGGA	AAATAATGCCCCACCAGCGA	142
appX	GTATTTACTTTGGTTCGTCGGCA	GGTCCAGCCATACCAACACA	71
*gyrA**	GTCGTGGCGGGAAAGGTAAA	CGGCTGGAGAAGCACAGAA	106
*frr**	GATCTTGGCCTGAACCCGAA	CTGCTTCACCACGAACGATTT	109

**housekeeping genes*
